# A Comprehensive Guide to Selecting and (Potentially) Replacing PACS: Navigating the Decision-Making Processes

**DOI:** 10.1007/s10278-025-01672-7

**Published:** 2025-09-19

**Authors:** Rajeev Nowrangi, Stacey M. Elangovan, Brian D. Coley, Eric J. Crotty, Arnold C. Merrow, Usha D. Nagaraj, Sara M. O’Hara, Susan N. Smith, Paula Bennett, Alexander J. Towbin

**Affiliations:** 1 Department of Radiology, Loma Linda University, Loma Linda, USA; 2 Department of Radiology, Cincinnati Children’s Hospital, Cincinnati, OH USA; 3 Department of Radiology, University of Cincinnati College of Medicine, Cincinnati, OH USA

**Keywords:** PACS, Imaging informatics, Procurement, Governance

## Abstract

Selecting a Picture Archiving and Communication System (PACS) is a strategic decision that impacts radiology workflow, communication, and operational efficiency. Despite its importance, there is limited guidance on structured approaches to PACS procurement. This study aims to describe a comprehensive, data-driven approach to evaluating and selecting a clinical PACS within a radiology department. A six-phase process was developed: team formation, expectation setting, background assessment, initial vendor assessment, virtual demonstrations, and final evaluation. A multidisciplinary committee identified key pillars and concepts for PACS functionality, which informed vendor evaluations through surveys, standardized demonstrations, and a detailed request for proposal. Quantitative and qualitative data were used at each phase to score vendors across multiple dimensions including usability, integration, performance, and cost. Eleven pillars and 236 concepts were defined and weighted to evaluate vendor solutions. Five vendors were shortlisted after an initial presentation. These vendors were invited to provide a virtual demonstration. Three vendors were then selected for onsite assessments using department-generated anonymized datasets. Comprehensive RFPs and cost analyses were incorporated into final evaluations. Ultimately, the incumbent vendor was selected with a recommendation for reevaluation in 3 years, guided by detailed assessment metrics and stakeholder feedback. This case study offers a potentially reproducible methodology for healthcare institutions evaluating PACS solutions. Emphasizing transparency, stakeholder engagement, and data-driven decision-making, the approach should be adaptable to other technology procurement efforts and scalable to smaller projects.

**Supplementary Information:**

The online version contains supplementary material available at 10.1007/s10278-025-01672-7.

## Introduction

In today’s digital healthcare landscape, Picture Archiving and Communication Systems (PACS) are indispensable. These systems are foundational to radiology department and hospital operations, enhancing workflow efficiencies, ensuring accurate patient records, and facilitating essential communication across diverse care settings. However, when PACS are poorly designed or overly complex, they can significantly increase the workload for radiologists and technologists, create substantial communication barriers, and lead to employee dissatisfaction and burnout [1, 2].

The selection of a PACS, therefore, is not just a technical decision but a strategic one that impacts every aspect of healthcare delivery within a radiology department [1, 2]. Given the significant disruptions and costs associated with replacing a PACS, the decision-making process must be comprehensive, measured, and forward-thinking [1–6]. Unfortunately, there is no one-size-fits-all method for healthcare software selection and procurement [1–3, 7–17]. The optimal approach likely depends on factors such as the system's complexity, its importance in day-to-day operations, the number of users and roles impacted by the software, and its cost.

This manuscript outlines our department’s approach to evaluating the PACS marketplace and selecting a vendor, aiming to offer a reproducible methodology for healthcare institutions facing similar technology procurement challenges. While detailed, we believe this approach can be adapted for selecting less complex software applications, offering a versatile framework that extends beyond the specific context of PACS.

## Overview of the PACS Selection Process

The PACS selection process detailed in this manuscript was implemented at a large, free-standing children’s hospital. The Radiology Department performs approximately 280,000 imaging studies annually across all radiologic specialties except breast imaging. Its digital archive dates to 2000 and contains over 5 million imaging studies. The department was operating with its second PACS, which replaced the initial system in 2010 after a decade of use. Despite mild dissatisfaction among radiologists with the current PACS, there was no overarching consensus for its replacement. Thus, the primary objective was not immediate replacement but a thorough evaluation of the market to determine if a newer PACS could improve departmental efficiency and workflow.

The department employes 41 faculty radiologists across 10 clinical sections, 253 technologists, and 11 IT professionals. In addition to these employees, multiple trainees work in the department and interact with the PACS including fellows, residents, and students. The hospital has more than 19,000 employees, most of whom have access to view imaging studies.

This hospital has a history of implementing enterprise imaging solutions across various departments and divisions, each with uniquely tailored workflows to optimize data and operational efficiency [18–21]. Although all radiology and select other departmental imaging studies initially pass through the radiology PACS, they are ultimately stored in a vendor-neutral archive (VNA), with most hospital personnel and all patients accessing these images via an enterprise viewer, rather than directly through the radiology PACS [18].

Given this complex imaging ecosystem, the scope of this project was defined to include only the clinical Radiology PACS for potential review and replacement. Other systems that were excluded from review included the research PACS, enterprise viewer, other specialty PACS (e.g., Cardiology and Pathology), the VNA, and reporting software. Our existing PACS contained an integrated worklist tool. While the current selection did not include stand-alone worklist tools, PACS without an integrated worklist were considered in scope. If one of these products were selected, the selection committee would then be tasked with identifying a worklist product.

The evaluation and selection process for the new PACS was structured into six distinct stages to be performed over the course of one fiscal year: team formation, expectation setting, background assessment, initial vendor assessment, virtual vendor presentations, and final assessment. Each stage was designed to not only progressively build departmental engagement but also to gather increasingly detailed information from the vendors related to their PACS offerings. Details of each stage and their strategic importance are discussed in the following sections. Because the selection process was to be carried out over the course of a fiscal year, formal budgetary approval was not obtained. However, a PACS replacement line item was included as part of the 5-year budget. As the project progressed, budgetary quotes were obtained from viable contenders to plan for the following fiscal year budget.

## Team Formation

In the first stage of the project, the PACS Selection Committee was formed, and the decision-making process was defined. The primary aim of this stage was to establish clear governance regarding the final decision on the PACS selection and to build a selection process that was transparent, inclusive, and aligned with the strategic needs of the department.

Initially, the Radiologist-in-Chief (REDACTED) appointed the Vice-Chief for Clinical Operations and Radiology Informatics (REDACTED) as Chair of the Committee and tasked it with making a recommendation regarding the clinical PACS. Although the Committee would make a recommendation, the Radiologist-in-Chief retained ultimate responsibility for the final decision.

To ensure broad representation and diverse perspectives within the committee, the Radiologist-in-Chief and the Committee Chair invited department members to volunteer. Ultimately, 10 members were chosen, each representing different facets of the department’s operations: each clinical section had at least one representative, technologist managers from radiography and ultrasound brought in the technologist perspective, and leaders from the department’s informatics team provided IT insights. A combined Pediatric Radiology/Imaging Informatics fellow was included as the fellow representative. A hospital procurement specialist was also involved as an ex officio member to assist in managing the process and interacting with vendors, although they did not participate in the final decision-making. Clinicians outside the Radiology Department were not included, given their minimal use of the Radiology PACS.

Once the team was established, the Committee Chair formed a leadership team consisting of himself, the IT leaders, the fellow representative, and the procurement specialist. This leadership team met weekly to meticulously plan and oversee the selection process. The key tasks led by the leadership team included the following:

1. Coordinating all communications, demonstrations, and visits with vendors.
2. Creating meeting agendas and essential team documents.
3. Developing vendor assessment surveys to capture detailed feedback.
4. Analyzing data from vendor assessments and generating reports to inform decision-making.
5. Standardizing the demonstrations process to ensure consistency in vendor presentations.
6. Authoring the request for proposal (RFP) document to elicit detailed information from vendors.
7. Analyzing responses to the RFP to evaluate vendors comprehensively.
8. Scheduling and attending meetings with vendors and their reference sites.
9. Providing regular updates to maintain transparency and engage the department.

## Expectation Setting

After forming the team, the project advanced into its second stage, where the committee convened to define its goals, identify potential outcomes, and establish expectations concerning the project's scope. This phase aimed to provide the committee with clear directives, foster a common understanding of participation expectations, and establish the rules for committee engagement.

The committee’s leadership drafted each element. These drafts served as the foundation for committee discussions, with all final decisions made by consensus.

### Goals of the PACS Selection Process:

1. Identify and prioritize elements of PACS critical for day-to-day clinical operations within the department.
2. Evaluate the current PACS marketplace.
3. Make a recommendation to the Radiologist-in-Chief regarding PACS by the end of the fiscal year.

### Potential Final Recommendations:

1. Stay with the current PACS vendor.
2. Select a new PACS vendor.
3. Retain the current PACS vendor but plan a re-evaluation after a short interval.

### Guiding Principles for the Selection Process and Committee Participation:

1. Transparency: Keep the department informed by documenting and sharing each decision and its rationale.
2. Openness: Solicit and incorporate feedback from non-committee members through surveys and open forums to ensure all perspectives are considered.
3. Honesty: Uphold integrity by providing accurate assessments of vendor capabilities and departmental needs.
4. Inquisitiveness: Promote a culture of curiosity where questions are encouraged to deepen understanding of technologies and processes.
5. Collaboration: Work synergistically across different departmental roles to ensure decisions benefit all stakeholders.
6. Dedication: Expect committed participation in all scheduled meetings and evaluations from each committee member.

### Vendor Assessment and Selection Process:

1. Background Assessment:
  • Goal: Identify essential PACS features for our department.
  • Method: Conduct surveys and engage in in-depth discussions within the Selection Committee to gather insights on necessary features.
  • Participants: Selection Committee (required) to provide foundational decisions, with the informatics team (optional) contributing specialized technical insights.
  • Timeline: Complete before the Radiological Society of North America (RSNA) Annual Meeting.
  • Commitment: Six 1-h meetings.
  Outcome: A prioritized list of features, a vendor scoring model based on identified features, and draft RFP questions designed to probe these areas.
2. Initial Vendor Assessment:
  • Goal: Evaluate the PACS marketplace to identify suitable vendors.
  • Method: Attend booth demonstrations at the RSNA meeting to interact with vendors and assess their offerings.
  • Participants: Selection leadership team (required) to ensure strategic alignment, with other committee members (optional) enriching the evaluation with their varied perspectives.
  • Timeline: Conduct during the RSNA.
  • Commitment: Ten 1.5- to 2-h demonstrations.
  • Outcome: Identification of five vendors that best meet the department’s criteria for further evaluation.
3. Vendor Presentations:
  • Goal: Narrow down suitable vendors.
  • Method: Host virtual vendor presentations to delve deeper into each vendor’s capabilities and fit with departmental needs.
  • Participants: Selection Committee (required) for core product assessment, informatics team, radiologist faculty members, and modality managers (all optional) to provide broader departmental input.
  • Timeline: January–March.
  • Commitment: Five 1.5-h presentations and two 1-h committee meetings to discuss findings.
  • Outcome: List of three final vendors for detailed RFP and on-site demonstrations.
4. Demonstrations and RFP:
  • Goal: Make a final recommendation to the Radiologist-in-Chief.
  • Method: Conduct standard evaluations during live demonstrations and thorough review of RFP responses to assess each vendor’s alignment with departmental requirements.
  • Participants: Selection Committee (required) for detailed evaluation, with the informatics team, departmental radiologists, and technologist managers (optional) offering less detailed feedback on user experience and technical compatibility.
  • Timeline: March–June.
  • Commitment: Three 1–2-h demonstrations and two 1-h committee meetings for deliberation.
  • Outcome: A final recommendation based on a holistic assessment of each vendor’s capabilities, alignment with departmental needs, and potential for long-term partnership.

## Background Assessment

During this stage, the PACS Selection Committee concentrated on identifying and evaluating the key elements critical for an optimal PACS within our department. The purpose of this stage was to start the Committee’s open discussion and demonstrate its decision-making process.

Initially, committee members were tasked with defining a list of “pillars,” or foundational elements that group feature sets within the PACS application. Examples of such pillars included “Usability and User Interface,” “Integration with other Systems,” and “Performance.” These pillars and concepts were similar to the dimensions and subdimensions described in previous PACS procurement articles [5]. Each committee member presented their proposed pillars at a subsequent meeting where these suggestions were collaboratively refined and merged into a final list.

Following the pillar identification, committee members developed a list of “concepts” for each pillar. These concepts represent specific feature sets crucial for the PACS functionality, such as “Hanging protocols” under “Usability and User Interface” or “First image load time” under “Performance.” The development of concept lists proceeded similarly to the pillars, with each member contributing detailed suggestions, which were then collaboratively refined.

The committee utilized two surveys to evaluate these elements further. The first survey required members to rank each pillar according to its importance to the department’s needs. The second survey asked members to rate each concept on a 5-point Likert scale, categorizing them as “1—Not Needed,” “3—Nice to Have,” and “5—Need to Have.”

### Outcome

The Selection Committee identified 11 pillars and 236 concepts. Pillars are included in Table 1. Concepts are included in Supplemental Table 1.

**Table 1:** Pillars important for PACS selection in our department

Pillar	Mean pillar rank^a^	Pillar rank score^b^
Usability and user interface	1.8	10.2
Workflows	3.2	8.8
Performance	3.3	8.7
Toolsets and other specialized functionality	4.1	7.9
Integration with other systems	5.8	6.2
Communication tools	6.2	5.8
System administration and support	6.4	5.6
Education	7.7	4.3
Interoperability	8.2	3.8
Technology	8.8	3.2
Procurement and implementation	10.5	1.5

^a^The mean pillar rank was calculated by determining the arithmetic mean of the pillar ranking from each committee member. A lower rank means that the pillar was determined to be more important

^b^The pillar rank score was calculated by the formula pillar rank score = 12 − mean pillar rank. A higher score means that the pillar was determined to be more important

The arithmetic mean ranking was calculated for each pillar. A pillar rank score was then calculated using the formula: pillar rank score = 12 − mean pillar rank. Using this formula, a higher score indicated that the corresponding pillar was of greater importance to the Committee. The pillar rank score served as a weighting factor during later steps of the selection process.

Each concept was rated by all Committee members. The mean rating per concept was calculated. The product of the pillar score and the concept rating were used to identify the most important features of a PACS.

**Fig. 1:**
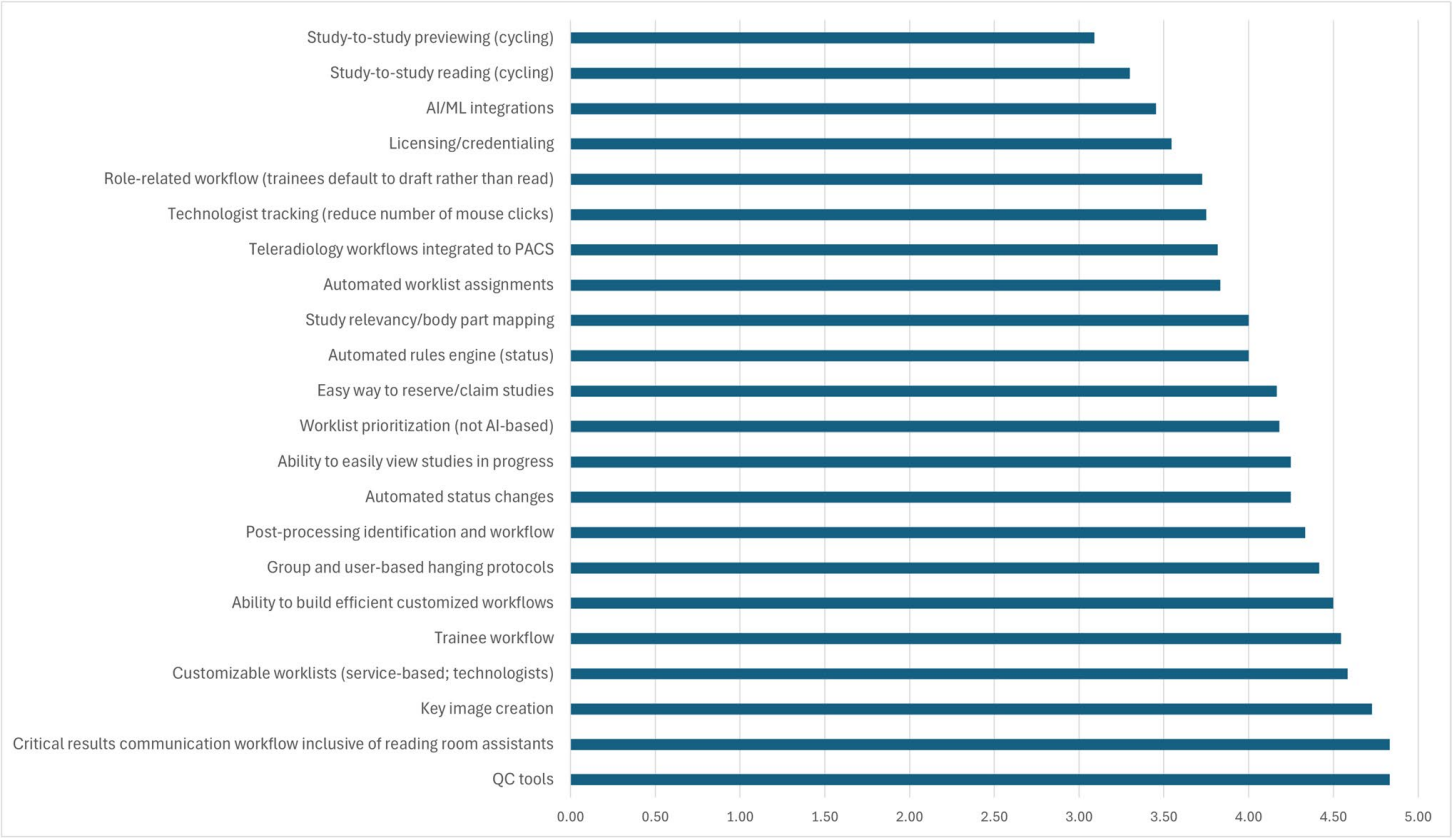
Bar chart showing the selection committee’s ratings of concepts related to the workflow pillar. Higher ratings are rated as more important than lower ratings

Bar charts showing the importance of each concept within each pillar were created (example in Fig. 1). All faculty radiologists were invited to a meeting where the ranked pillars and rated concepts were discussed. The Committee chair led the faculty discussion inviting general feedback on the process and early outcomes. Meeting minutes containing the ranked pillars and rated concepts were subsequently distributed to the faculty via email.

## Initial Vendor Assessment

Once the essential PACS features were identified, the Committee’s leadership conducted a survey of the PACS marketplace to determine potential vendors, understand current consumer sentiment, and identify a list of vendors best suited to meet the department’s needs.

The leadership engaged in informal discussions with peers at various institutions who had recently made PACS decisions, noting both the vendors they replaced and those selected. These conversations provided insights into current trends and vendor performance. Additionally, the leadership reviewed product evaluations from an independent healthcare technology research firm [KLAS, Park City, UT] to supplement their understanding of vendor standings and capabilities.

Based on this preliminary research, 10 potential PACS vendors were identified. Meetings and demonstrations, lasting between 1.5 and 2 h, were arranged with nine of these vendors at the RSNA annual meeting, with a tenth vendor available for an impromptu session. These demonstrations were allowed to progress in a free-form manner based on the vendor. However, the team’s questions were guided by the previously identified pillars. Specifically, they asked to see features related to radiologist workflow, technologist workflow, quality control (QC) editing, radiologist tool sets, multi-disciplinary conference tools, interruption workflow, hanging protocols, teaching files, communication tools, system administrator tools, and search functions. The team also asked questions related to infrastructure, system architecture, system readiness for cloud storage, and artificial intelligence (AI).

### Outcome

Post-demonstration, attendees conducted an informal debriefing to discuss their impressions and any standout features or concerns. This led to a consensus-based ranking of the vendors and a list of pros and cons of each vendor. Initially, the team identified a clear top four, a distinct fifth, and a sixth-ranked vendor, although there was some debate about the exact order of the top four.

After the RSNA meeting, the Committee leadership discussed the demonstrations and ranking with the selection committee. The Committee was asked if they would like to be presented with a slate of 4, 5, or 6 vendors knowing that there was a clear separation between vendors ranked between 4, 5, and 6. They were asked to make this decision without knowing the vendors rank order or the pros and cons of each vendor.

After a brief discussion, the Committee decided to move forward with the top five vendors on their shortlist. The pros and cons of the vendors ranked 6–10 were shared with the selection team. A vendor-anonymized example of this document is included in Table 2. The pros and cons of vendors ranked 1–5 were not shared as not to bias further evaluation. Of note, the incumbent vendor was included on the shortlist as one of the top five vendors.

**Table 2:** Sample pros and cons of a PACS vendor that did not meet criteria to move forward for further assessment

Pros	Cons
Good communication tools	Separate applications are needed for quality control and administrator functionality
Ability to build a workflow for reading room assistants	Limited ability to create teaching files or perform multi-disciplinary conferences
Good interruption workflow	Reliant on worklist application that department had already stated their displeasure
Good portal for system administrators	Poor search functionality
Good technologist quality control workflow	Bad user interface

## Virtual Vendor Assessment

With the shortlist of vendors established, the committee proceeded to the virtual vendor assessment phase. The primary goal was to understand the differences in vendor platforms, focusing particularly on the workflows for radiologists, technologists, and system administrators.

To accomplish this goal, the selection committee developed a standardized demonstration format that covered 23 key topics (Table 3) derived from the previously identified pillars and concepts. This format ensured that each vendor presentation would address areas such as system usability, integration capabilities, and technical support. Each vendor, except for the incumbent, was instructed to adhere strictly to this format. Due to the committee’s existing familiarity with the incumbent vendor’s system, they were specifically asked to present recent updates, address any committee-identified feature gaps, and concentrate on technologist and administrator workflows.

**Table 3:**
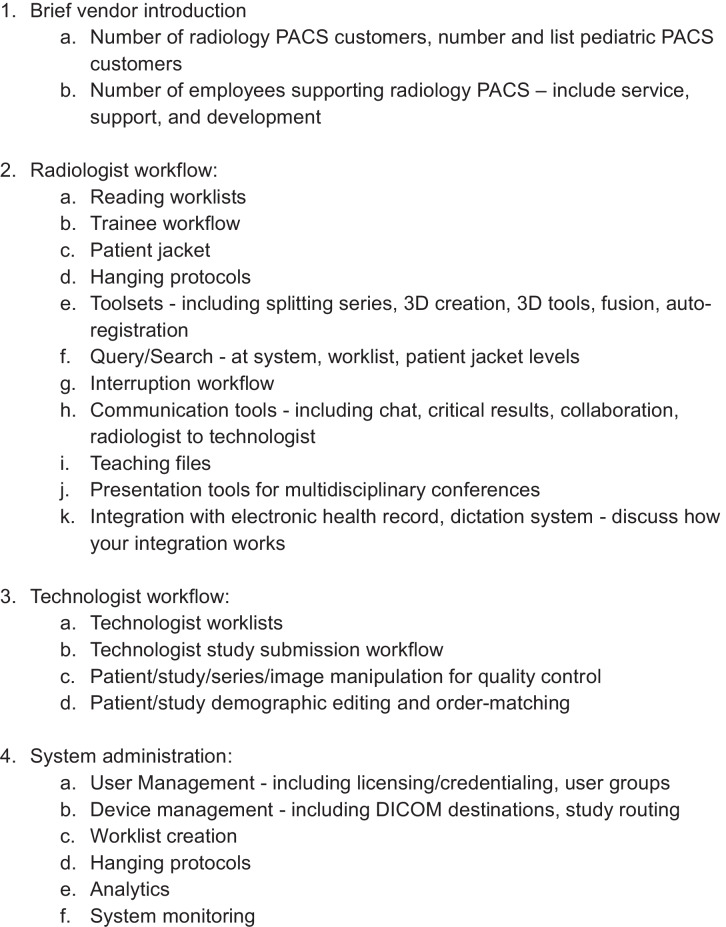
Standard virtual demonstration format provided to each vendor

Each vendor was allocated a 90-min slot for their virtual presentation, recorded for thorough review and analysis. Attendance was mandatory for selection committee members, with a broader invitation extended to all radiologists and technologist managers within the department. To promote active engagement, committee members were encouraged to ask live questions while other attendees contributed via chat.

### Assessment

Prior to the demonstrations, a standard assessment survey (Supplemental Document 1) was created. The survey was designed to evaluate each vendor against the committee’s established pillars and concepts. Each survey element was rated on a 1–5 modified Likert scale. Additionally, Committee members were asked to provide an overall system score ranging from 0 to 100. Each member of the committee was asked to complete the entire assessment for each vendor. The selection committee was reminded that they should think beyond their role (radiologist, technologist, administrator) to ensure that the best PACS for the department would be selected.

After all of the demonstrations were complete, each Committee member was asked to complete a second survey ranking the vendors from first to fifth based on their overall impressions.

### Outcome of Virtual Demonstrations

The survey results were compiled and analyzed by the Committee Chair after all responses were collected, to prevent any interim bias. The analysis focused on several metrics:

The metrics assessed included.

1. Mean overall score (potential range 1–100, higher is better)
2. Mean sum of all rated elements (each rated on a 1–5 scale; total sum range 21–105; higher is better)
3. Mean of each individual rating (each rated on a 1–5 scale; average range 1–5, higher is better)
4. Sum of weighted scores (each weighted score was calculated by determining the product of the relevant pillar score, the relevant concept score, and the average 1–5 rating; all weighted scores were summed to create the sum of weighted scores. Note that if more than one concept was relevant, the highest scored concept was selected for weighting; higher is better)
5. Mean PACS ranking (potential range 1–5, lower is better)

These metrics above were used to rank each vendor. In addition, the mean ranking of all rankings was calculated. The results of each assessment are included in Table 4^4^. Note that the incumbent vendor is displayed in this table as vendor 5.

The selection committee convened to debrief and discuss the demonstration outcomes. This discussion helped to confirm the survey findings, particularly highlighting the performances of vendors 4 and 5. They noted that the incumbent vendor’s demonstration was difficult to assess due to the different script, the new product components that were shown, and their familiarity with the PACS.

**Table 4:**
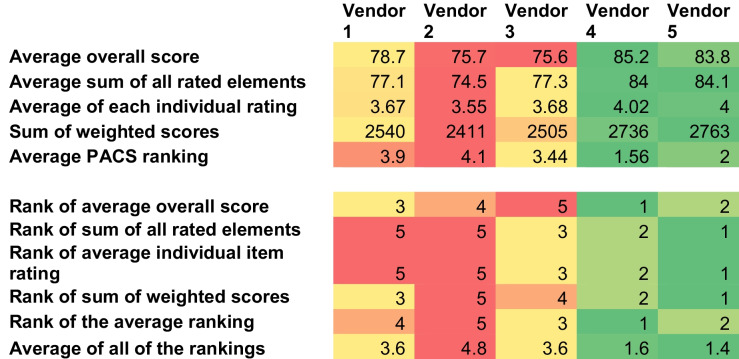
Heat map showing the results of selection committee assessment after virtual demonstrations

Heat map scale: Green items represent a highly rated metric, red scores represent a poorly rated metric.

Given the close rankings between certain vendors, the committee debated the optimal number of vendors to advance to the final assessment phase. Ultimately, it was decided to invite the top three vendors. Because there was a tie for third place (between vendors 1 and 3), the committee voted to determine which of the two tied vendors should advance to the final round. Vendor 3 was selected through this process. This decision, along with the supporting data and rationale, was shared with the entire faculty through a preparatory video that set the stage for a faculty meeting discussion.

## Final Assessment

This stage represented the culmination of the PACS selection process. This phase was designed to provide a comprehensive evaluation of the top three vendors through a series of onsite demonstrations, in-depth RFP assessments, and cost analyses. The objective was for the committee and department to scrutinize each system's functionality and alignment with the department's specific needs.

### Onsite Demonstrations

The three final vendors were each invited to perform a 3-day onsite demonstration that would allow the Committee and other members of the department to assess the features of the PACS.

All radiologists and modality managers were invited to attend the demonstrations. A sign-up tool was used to ensure that demonstrations were not over-crowded. The leadership team worked with the radiologist’s administrative assistants to ensure that each member of the selection committee was scheduled to attend each demonstration. The Radiology department leadership made the PACS selection a priority. Radiologists were encouraged to help each other attend the demonstrations and members of the selection committee were prioritized for office time on the day of onsite demonstrations.

Prior to the demonstration, the Radiology informatics team prepared an anonymized imaging dataset. Each vendor was asked to sign a business associate agreement so that data could be shared safely. After signing the document, each vendor was given the data and asked to load it onto their system so that attendees could see how the system would work using internal real-world data. Data was selected to highlight potential challenges including patients with more than 1000 comparison studies, studies with more than 20,000 images, studies with more than 1000 series, studies with mixed modalities, and studies with atypical data types (e.g., enhanced DICOM).

Vendors were responsible for setting up their systems and orchestrating the flow of their presentations. Their teams, which varied in size from two to eight individuals, adapted the demonstrations to the interests and questions of the attending radiologists and technologists, who participated in small groups of one to three people to foster a more interactive and personalized experience. All demonstrations were held in a reading room designed for testing and training. The radiologists could adjust the conditions to their liking, like clinical reading rooms.

### Assessment of Onsite Demonstrations

The selection committee deliberated on the most effective method to evaluate the different PACS. They considered several approaches:

1. Utilizing the same assessment form as used in previous phases.
2. Modifying the assessment form so that evaluations were based on roles.
3. Assigning specific system features to individuals to enable consistent evaluation across vendors.

Ultimately, the committee opted for the second approach, modifying the original survey to cater to the specific perspectives of radiologists, technologists, and administrators. This adaptation included more open-ended questions to capture feedback on system usability and functionality, and additional rating options for radiologists to assess various toolsets.

Like before, each element was rated on a 5-point Likert scale. The Committee members were also asked to provide an overall assessment score between 1 and 100. In addition to the selection committee, non-committee members were also invited to provide their assessments. Their evaluations included open-ended questions to express opinions and an overall assessment on the same 0 to 100 scale.

After the surveys were completed, the committee chair categorized the open-ended responses to assess the most liked and most disliked features of each PACS. The categories and items mentioned more than one time are included in Table 5.

**Table 5:** Qualitative assessment of the positives and negatives features of each PACS vendor following onsite demonstrations

Vendor 3			
Positive features	Mentions	Negative features	Mentions
Toolsets	5	User interface	3
Trainee workflow	2	Worklist	3
Hanging protocols	2	Interruption workflow	2
Auto registration	2	Patient jacket	2
Configuration	2	AI functionality	2
Vendor 4			
Positive features	Mentions	Negative features	Mentions
3D Functionality	7	Interruption workflow	4
Toolsets	6	System architecture	3
Conferences	5	Worklist	2
User interface	3	Lack of database access	2
Patient jacket	2	Reliance on vendor	2
Integration	2	QC editing	2
AI integration	2		
Vendor 5			
Positive features	Mentions	Negative features	Mentions
Workflow	5	Toolsets	4
Familiarity	4	Conferences	3
Worklists	3	3D functionality	2
Macros	2	Performance	2
Chat	2	User interface	2
System architecture	2		

*AI* artificial intelligence, *3D* three dimensional, *QC* quality control

### Request for Proposal

The leadership team authored an RFP document (Supplement Document 2). This document was extensive, containing approximately 920 targeted questions that covered a wide range of topics crucial for assessing each system’s suitability [12, 22–26]. These topics included pricing, data migration capabilities, system architecture, required hardware and software, ongoing support and maintenance, implementation and training processes, system upgrades, administration, and workflows specific to various roles such as general users, reading room assistants, technologists, and radiologists.

The vendors were given 1 week to review the RFP and submit any clarifying questions. The leadership team then responded to these inquiries the following week, and the clarifications were shared with all vendors to maintain transparency and fairness in the bidding process. Vendors had 4 weeks total to respond to the RFP from the time it was originally sent. As part of their responses, vendors were also required to include detailed projections of the 5-year total cost of ownership for their system.

### Request for Proposal Assessment

All vendors responded to the RFP. Each vendor’s response included multiple files beyond the original documents. The total number of files provided by vendors ranged from 4 to 57.

The committee chair independently reviewed each response, applying a structured scoring system to ensure a semi-objective evaluation. Scores were assigned based on the following scale:

- No score: Applied where no response was provided or the question was not applicable (often due to conditional if/then scenarios).
- 0 (Does Not Meet Expectations): Used for responses that failed to meet the baseline requirements or did not provide the requested features.
- 1 (Meets Expectations): Assigned to responses that adequately met the RFP requirements.
- 2 (Exceeds Expectations): Given to responses that surpassed what was asked, indicating a higher value or innovation beyond the basic criteria.

The average score for each vendor was calculated and then multiplied by 100 to create an RFP response score. Items not scored were not included when the average was calculated. A score of 100 or more indicated that responses generally met or exceeded expectations, while scores below 100 suggested deficiencies in meeting the requirements.

The RFP responses were comparatively ranked based on the quality of key features. In cases where differences were meaningful, rankings were assigned to highlight those vendors that provided superior responses. If vendors tied in their rankings on features, they were given the same score, and adjustments were made to the ranks of subsequent vendors accordingly. The RFP comparison score was calculated for each vendor by summing up their ranks across all features, with a lower score indicating a more favorable evaluation.

### Cost Comparison

Each vendor responded with a quote so that a 5-year total cost of ownership could be calculated. Two of the three remaining vendors provided a cost per study quote in case of overages to the contracted study volume.

### Outcome of Onsite Demonstrations and RFP Scoring

The comprehensive analysis of the assessment data was conducted by the committee chair. The analysis incorporated various metrics to provide a multifaceted view of each vendor’s performance and compatibility with the department’s needs.

The metrics assessed included the following:

1. Mean overall score from Selection Committee members (1–100; higher is better)
2. Median overall score from Selection Committee members (1–100; higher is better)
3. Sum of ratings from Selection Committee Members (20–100; higher is better)
4. Average rating score from Committee Members (1–5; higher is better)
5. Sum of weighted scores from Committee Members [Aggregate of weighted scores, calculated by multiplying the relevant pillar scores, the concept scores, and their average ratings] (higher is better)
6. RFP comparison score (lower is better)
7. RFP response score (higher is better)
8. Other reviewers’ mean overall score (1–100; higher is better)
9. Other reviewers’ median overall score (1–100; higher is better)
10. 5-Year total cost of ownership
11. Cost per study

The results of each assessment and the ranking of each assessment are included in Table 6.

**Table 6:**
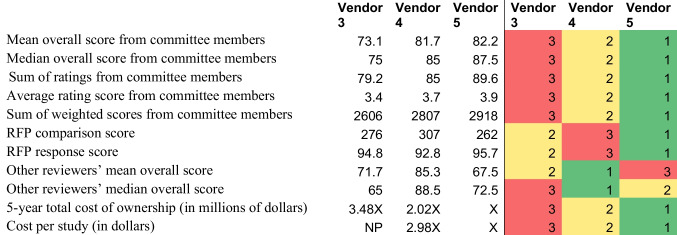
Results of assessment after different modes of final assessment

Heat map scale: green items represent a highly rated metric, red scores represent a poorly rated metric. Relative pricing data is included rather than specifics. The index price is included as X with comparators displayed as multiples of X. *RFP* request for proposal, *NP* not provided

### Other Methods of Assessment

In addition to the structured onsite demonstrations and RFP assessments, the leadership team employed other methods to further validate vendor capabilities and reliability.

#### Reference Calls

The leadership team conducted reference calls with customers of the two non-incumbent vendors to gain insights into real-world applications and performance. To ensure a balanced view, the leadership team spoke with one customer identified via their personal network and another customer suggested by the vendor. These discussions typically involved a PACS administrator and a radiologist from the reference sites, who provided detailed feedback on various aspects of their PACS. At the outset of the call, the leadership team asked call participants for any potential conflicts of interest. During the subsequent conversation, questions were asked to determine implementation success, vendor support and responsiveness, system architecture, system performance, and downtimes.

#### In-person Meetings

The team also leveraged the Society for Imaging Informatics in Medicine (SIIM) Annual Meeting to engage directly with the vendors. These interactions focused on addressing specific concerns and deficiencies noted during earlier assessments. Discussion topics included system architecture, business continuity, cloud and AI readiness, total cost, and the potential relationship with the department.

## Final Decision

The Selection Committee convened twice to finalize their recommendation concerning the PACS vendor. In the first meeting, the committee conducted a debrief of the onsite demonstrations. They validated the findings of the qualitative assessment and noted inconsistencies in how vendors handled departmental data integration during their demonstrations:

- One vendor failed to incorporate any provided data into their system.
- Another vendor included the data but only displayed it upon specific request.
- The third vendor exclusively used the provided departmental data, which limited their ability to demonstrate other system features.

After the debrief, the committee chair presented a detailed analysis of the RFP scoring, including examples of responses and an overview of how each vendor was evaluated across various metrics. This presentation was followed by a review of the assessment data and a comparison of RFP responses and cost analyses.

During the discussions, committee members requested further details on the vendors’ cloud readiness and AI integration capabilities. These topics were thoroughly discussed in the second meeting, incorporating feedback from the SIIM Annual Meeting.

After careful consideration, the committee recommended continuing with the incumbent vendor despite some identified deficiencies. They advised that this decision be re-evaluated in 3 years to ensure the vendor addresses these shortcomings effectively. This recommendation was unanimously agreed upon by all committee members.

Based on the Committee’s recommendation and his own experience during the selection and demonstration process, the Radiologist-in-Chief accepted the committee’s recommendation as the final decision.

## Conclusion

The PACS selection process at our institution serves as a case study describing a systematic and comprehensive approach for technology procurement. From the onset, this project was designed to provide the evaluation team with increasing levels of information and engagement, aligning with each phase of the selection.

We believe that one of the most important steps of our selection process was in identifying our guiding principles of transparency, openness, honesty, inquisitiveness, collaboration, and dedication. These principles shaped the framework within which we operated and ensured that each step remained aligned to the department’s strategic goals rather than the individual preferences of selection committee members.

Data played a pivotal role in our selection process, from the detailed assessment of vendor capabilities during onsite demonstrations and virtual assessments to the critical analysis of RFP responses and cost comparisons including associated financial, time, personnel, and infrastructure expenses. The use of quantitative metrics and qualitative feedback allowed us to maintain a high level of transparency and provided a solid foundation to guide our decisions and explain them to peers within the department. This became evident near the end of the process when there was a discrepancy between selection committee members and others within the department.

While our process was detailed and rigorous, we believe that it can be adapted and used for less complex technology solutions. Smaller scale projects may not require as extensive a process, allowing for adjustments in the depth of evaluation and the breadth of stakeholder engagement. Similar frameworks have previously been described for technology procurement [1–16, 27].

In summary, our PACS selection process highlighted the importance of structured evaluation, the power of data-driven decision-making, and the effectiveness of maintaining clear guiding principles. It has established a benchmark for how we select key technology, ensuring that the solution we choose is not just adequate but optimal for our department and institution’s needs.

## Supplementary Information

Below is the link to the electronic supplementary material.

ESM 1 Supplemental Document 1 (DOCX 18.6 KB)

ESM 2 Supplemental Document 2 (XLSX 72.3 KB)

ESM 3 Supplemental Table 1 (XLSX 367 KB)
