# Supplementary material for: A Comprehensive Guide to Selecting and (Potentially) Replacing PACS: Navigating the Decision-Making Processes
Source: J Imaging Inform Med. 2025 Sep 19;39(3):1992–2003. doi: 10.1007/s10278-025-01672-7 (PMC13230394; doi:10.1007/s10278-025-01672-7)
Supplement: Supplementary file 1 — Supplemental Document 1 (DOCX 18.6 KB) [file 10278_2025_1672_MOESM1_ESM.docx]

PACS Virtual Vendor Demo Evaluation

1. Name: _______________________
2. Job Role:

- Radiologist
- Technologist
- IT Professional

1. Vendor

- Vendor 1
- Vendor 2
- Vendor 3
- Vendor 4
- Vendor 5

1. **How would you rate the following items related to radiologist workflow?** When performing your evaluation consider ease-of-use, functionality, and completeness of the feature set.

|  | Very poor | Poor | Average | Good | Excellent |
| --- | --- | --- | --- | --- | --- |
| Reading worklists |  |  |  |  |  |
| Trainee workflow |  |  |  |  |  |
| Patient jacket |  |  |  |  |  |
| Hanging protocols |  |  |  |  |  |
| Toolsets (including series splitting, 3D creation, 3D tools, fusion, auto-registration) |  |  |  |  |  |
| Query/search (system, worklist, patient jacket) |  |  |  |  |  |
| Interruption Workflow |  |  |  |  |  |
| Communication tools including chat, critical results notification, collaboration, radiologist --> technologist) |  |  |  |  |  |
| Teaching files |  |  |  |  |  |
| Presentation tools for multidisciplinary conferences |  |  |  |  |  |
| Integration with EHR, dictation system |  |  |  |  |  |

1. **How would you rate the following items related to technologist workflow?** When performing your evaluation consider ease-of-use, functionality, and completeness of the feature set.

|  | Very poor | Poor | Average | Good | Excellent |
| --- | --- | --- | --- | --- | --- |
| Tech worklists |  |  |  |  |  |
| Tech study submission workflow |  |  |  |  |  |
| Patient/Study/Series/Image manipulation |  |  |  |  |  |
| Demographic editing and order matching |  |  |  |  |  |

1. **How would you rate the following items related to administrator workflow?** When performing your evaluation consider ease-of-use, functionality, and completeness of the feature set.

|  | Very poor | Poor | Average | Good | Excellent |
| --- | --- | --- | --- | --- | --- |
| User management (including licensing/credentialing, user groups) |  |  |  |  |  |
| Device management (including DICOM destinations, study routing) |  |  |  |  |  |
| Worklist creation |  |  |  |  |  |
| Hanging protocols |  |  |  |  |  |
| Analytics |  |  |  |  |  |
| System monitoring |  |  |  |  |  |
